# DisProt in 2022: improved quality and accessibility of protein intrinsic disorder annotation

**DOI:** 10.1093/nar/gkab1082

**Published:** 2021-11-25

**Authors:** Federica Quaglia, Bálint Mészáros, Edoardo Salladini, András Hatos, Rita Pancsa, Lucía B Chemes, Mátyás Pajkos, Tamas Lazar, Samuel Peña-Díaz, Jaime Santos, Veronika Ács, Nazanin Farahi, Erzsébet Fichó, Maria Cristina Aspromonte, Claudio Bassot, Anastasia Chasapi, Norman E Davey, Radoslav Davidović, Laszlo Dobson, Arne Elofsson, Gábor Erdős, Pascale Gaudet, Michelle Giglio, Juliana Glavina, Javier Iserte, Valentín Iglesias, Zsófia Kálmán, Matteo Lambrughi, Emanuela Leonardi, Sonia Longhi, Sandra Macedo-Ribeiro, Emiliano Maiani, Julia Marchetti, Cristina Marino-Buslje, Attila Mészáros, Alexander Miguel Monzon, Giovanni Minervini, Suvarna Nadendla, Juliet F Nilsson, Marian Novotný, Christos A Ouzounis, Nicolás Palopoli, Elena Papaleo, Pedro José Barbosa Pereira, Gabriele Pozzati, Vasilis J Promponas, Jordi Pujols, Alma Carolina Sanchez Rocha, Martin Salas, Luciana Rodriguez Sawicki, Eva Schad, Aditi Shenoy, Tamás Szaniszló, Konstantinos D Tsirigos, Nevena Veljkovic, Gustavo Parisi, Salvador Ventura, Zsuzsanna Dosztányi, Peter Tompa, Silvio C E Tosatto, Damiano Piovesan

**Affiliations:** Institute of Biomembranes, Bioenergetics and Molecular Biotechnologies, National Research Council (CNR-IBIOM), Bari, Italy; Department of Biomedical Sciences, University of Padova, Padova, Italy; Structural and Computational Biology Unit, European Molecular Biology Laboratory, Heidelberg 69117, Germany; Department of Biomedical Sciences, University of Padova, Padova, Italy; Department of Biomedical Sciences, University of Padova, Padova, Italy; Institute of Enzymology, Research Centre for Natural Sciences, Budapest 1117, Hungary; Instituto de Investigaciones Biotecnológicas (IIBiO-CONICET), Universidad Nacional de San Martín, Av. 25 de Mayo y Francia, CP1650 Buenos Aires, Argentina; Department of Biochemistry, Eötvös Loránd University, Pázmány Péter stny 1/c, Budapest H-1117, Hungary; VIB-VUB Center for Structural Biology, Vlaams Instituut voor Biotechnology, Brussels, Belgium; Structural Biology Brussels (SBB), Bioengineering Sciences Department, Vrije Universiteit Brussel (VUB), Brussels, Belgium; Institut de Biotecnologia i Biomedicina, Universitat Autònoma de Barcelona, Barcelona, Spain; Departament de Bioquímica i Biologia Molecular, Universitat Autònoma de Barcelona, Barcelona, Spain; Institut de Biotecnologia i Biomedicina, Universitat Autònoma de Barcelona, Barcelona, Spain; Departament de Bioquímica i Biologia Molecular, Universitat Autònoma de Barcelona, Barcelona, Spain; Institute of Enzymology, Research Centre for Natural Sciences, Budapest 1117, Hungary; VIB-VUB Center for Structural Biology, Vlaams Instituut voor Biotechnology, Brussels, Belgium; Structural Biology Brussels (SBB), Bioengineering Sciences Department, Vrije Universiteit Brussel (VUB), Brussels, Belgium; Institute of Enzymology, Research Centre for Natural Sciences, Budapest 1117, Hungary; Cytocast Kft., Vecsés, Hungary; Department of Woman and Child Health, University of Padova, Padova, Italy; Pediatric Research Institute, Città della Speranza, Padova, Italy; Science for Life Laboratory, Department of Biochemistry and Biophysics, Stockholm University, 171 21 Solna, Sweden; Biological Computation & Process Laboratory, Chemical Process & Energy Resources Institute, Centre for Research & Technology Hellas, Thermi, Thessalonica 57001, Greece; Institute of Cancer Research, Chester Beatty Laboratories, 237 Fulham Rd, Chelsea, London, UK; Laboratory for Bioinformatics and Computational Chemistry, Vinča Institute of Nuclear Sciences, National Institute of the Republic of Serbia, University of Belgrade, 11000Belgrade, Serbia; Structural and Computational Biology Unit, European Molecular Biology Laboratory, Heidelberg 69117, Germany; Institute of Enzymology, Research Centre for Natural Sciences, Budapest 1117, Hungary; Science for Life Laboratory, Department of Biochemistry and Biophysics, Stockholm University, 171 21 Solna, Sweden; Department of Biochemistry, Eötvös Loránd University, Pázmány Péter stny 1/c, Budapest H-1117, Hungary; Swiss-Prot group, SIB Swiss Institute of Bioinformatics, Geneva, Switzerland; Institute for Genome Sciences, University of Maryland School of Medicine 670 W. Baltimore St., Baltimore, MD 21201, USA; Instituto de Investigaciones Biotecnológicas (IIBiO-CONICET), Universidad Nacional de San Martín, Av. 25 de Mayo y Francia, CP1650 Buenos Aires, Argentina; Bioinformatics Unit, Fundación Instituto Leloir, Buenos Aires, C1405BWE, Argentina; Institut de Biotecnologia i Biomedicina, Universitat Autònoma de Barcelona, Barcelona, Spain; Departament de Bioquímica i Biologia Molecular, Universitat Autònoma de Barcelona, Barcelona, Spain; Faculty of Information Technology and Bionics, Pázmány Péter Catholic University, Práter u. 50/A, 1083 Budapest, Hungary; Cancer Structural Biology, Danish Cancer Society Research Center, Strandboulevarden 49, 2100 Copenhagen, Denmark; Department of Woman and Child Health, University of Padova, Padova, Italy; Pediatric Research Institute, Città della Speranza, Padova, Italy; Lab. Architecture et Fonction des Macromolécules Biologiques (AFMB), UMR 7257, Aix Marseille University and Centre National de la Recherche Scientifique (CNRS), 163 Avenue de Luminy, Case 932, 13288, Marseille, France; Instituto de Biologia Molecular e Celular (IBMC), Universidade do Porto, 4200-135 Porto, Portugal; Instituto de Investigação e Inovação em Saúde (i3S), Universidade do Porto, 4200-135 Porto, Portugal; Cancer Structural Biology, Danish Cancer Society Research Center, Strandboulevarden 49, 2100 Copenhagen, Denmark; Departamento de Ciencia y Tecnología, Universidad Nacional de Quilmes - CONICET, Bernal, Buenos Aires B1876BXD, Argentina; Bioinformatics Unit, Fundación Instituto Leloir, Buenos Aires, C1405BWE, Argentina; VIB-VUB Center for Structural Biology, Vlaams Instituut voor Biotechnology, Brussels, Belgium; Structural Biology Brussels (SBB), Bioengineering Sciences Department, Vrije Universiteit Brussel (VUB), Brussels, Belgium; Department of Biomedical Sciences, University of Padova, Padova, Italy; Department of Biomedical Sciences, University of Padova, Padova, Italy; Institute for Genome Sciences, University of Maryland School of Medicine 670 W. Baltimore St., Baltimore, MD 21201, USA; Lab. Architecture et Fonction des Macromolécules Biologiques (AFMB), UMR 7257, Aix Marseille University and Centre National de la Recherche Scientifique (CNRS), 163 Avenue de Luminy, Case 932, 13288, Marseille, France; Dep. of Cell Biology, Faculty of Science, Vinicna 7, 128 43, Prague, Czech Republic; Biological Computation & Process Laboratory, Chemical Process & Energy Resources Institute, Centre for Research & Technology Hellas, Thermi, Thessalonica 57001, Greece; Biological Computation & Computational Biology Group, Artificial Intelligence & Information Analysis Lab, Department of Computer Science, Aristotle University of Thessalonica, Thessalonica 54124, Greece; Departamento de Ciencia y Tecnología, Universidad Nacional de Quilmes - CONICET, Bernal, Buenos Aires B1876BXD, Argentina; Cancer Structural Biology, Danish Cancer Society Research Center, Strandboulevarden 49, 2100 Copenhagen, Denmark; Cancer Systems Biology, Section for Bioinformatics, Department of Health and Technology, Technical University of Denmark, Lyngby, Denmark; Instituto de Biologia Molecular e Celular (IBMC), Universidade do Porto, 4200-135 Porto, Portugal; Instituto de Investigação e Inovação em Saúde (i3S), Universidade do Porto, 4200-135 Porto, Portugal; Science for Life Laboratory, Department of Biochemistry and Biophysics, Stockholm University, 171 21 Solna, Sweden; Bioinformatics Research Laboratory, Department of Biological Sciences, University of Cyprus, Nicosia, Cyprus; Institut de Biotecnologia i Biomedicina, Universitat Autònoma de Barcelona, Barcelona, Spain; Departament de Bioquímica i Biologia Molecular, Universitat Autònoma de Barcelona, Barcelona, Spain; Department of Cell Biology, Faculty of Science, Charles University, BIOCEV, Prague , Czech Republic; Departamento de Ciencia y Tecnología, Universidad Nacional de Quilmes - CONICET, Bernal, Buenos Aires B1876BXD, Argentina; Departamento de Ciencia y Tecnología, Universidad Nacional de Quilmes - CONICET, Bernal, Buenos Aires B1876BXD, Argentina; Institute of Enzymology, Research Centre for Natural Sciences, Budapest 1117, Hungary; Science for Life Laboratory, Department of Biochemistry and Biophysics, Stockholm University, 171 21 Solna, Sweden; Department of Biochemistry, Eötvös Loránd University, Pázmány Péter stny 1/c, Budapest H-1117, Hungary; European Molecular Biology Laboratory, European Bioinformatics Institute (EMBL-EBI), Wellcome Genome Campus, Hinxton, UK; Laboratory for Bioinformatics and Computational Chemistry, Vinča Institute of Nuclear Sciences, National Institute of the Republic of Serbia, University of Belgrade, 11000Belgrade, Serbia; Departamento de Ciencia y Tecnología, Universidad Nacional de Quilmes - CONICET, Bernal, Buenos Aires B1876BXD, Argentina; Institut de Biotecnologia i Biomedicina, Universitat Autònoma de Barcelona, Barcelona, Spain; Departament de Bioquímica i Biologia Molecular, Universitat Autònoma de Barcelona, Barcelona, Spain; ICREA, Barcelona, Spain; Department of Biochemistry, Eötvös Loránd University, Pázmány Péter stny 1/c, Budapest H-1117, Hungary; Institute of Enzymology, Research Centre for Natural Sciences, Budapest 1117, Hungary; VIB-VUB Center for Structural Biology, Vlaams Instituut voor Biotechnology, Brussels, Belgium; Structural Biology Brussels (SBB), Bioengineering Sciences Department, Vrije Universiteit Brussel (VUB), Brussels, Belgium; Department of Biomedical Sciences, University of Padova, Padova, Italy; Department of Biomedical Sciences, University of Padova, Padova, Italy

## Abstract

The Database of Intrinsically Disordered Proteins (DisProt, URL: https://disprot.org) is the major repository of manually curated annotations of intrinsically disordered proteins and regions from the literature. We report here recent updates of DisProt version 9, including a restyled web interface, refactored Intrinsically Disordered Proteins Ontology (IDPO), improvements in the curation process and significant content growth of around 30%. Higher quality and consistency of annotations is provided by a newly implemented reviewing process and training of curators. The increased curation capacity is fostered by the integration of DisProt with APICURON, a dedicated resource for the proper attribution and recognition of biocuration efforts. Better interoperability is provided through the adoption of the Minimum Information About Disorder (MIADE) standard, an active collaboration with the Gene Ontology (GO) and Evidence and Conclusion Ontology (ECO) consortia and the support of the ELIXIR infrastructure.

## INTRODUCTION

Whereas our traditional view of protein function is rooted in the model of proteins assuming a stable structure, a well-defined 3D fold, it is now >20 years since the concept of structural disorder of proteins has been proposed (1,2). The existence and functional importance of intrinsically disordered proteins*/*regions (IDPs*/*IDRs) is now generally accepted (3), with >1500 PubMed publications mentioning disordered proteins every year.

The prediction of protein disorder from sequence, for example, has always been an area of continuous activity. Recently, it has received a boost with the establishment of the Critical Assessment of Protein Intrinsic Disorder prediction (CAID) experiment as a community-wide blind test to compare state-of-the-art approaches to predict disorder (4). As new disorder prediction methods keep emerging (5–7), CAID takes on monitoring the field in real time, aiming to establish dependable standards. This ambition has a special caveat, as predicting and identifying regions in IDPs/IDRs that engage in functional interactions remains a significant challenge (6,8).

The prediction of such functional regions may support and inspire dedicated experimental approaches. The Eukaryotic Linear Motif (ELM) database is a primary repository of such data (9). Of similar ambition pursued along different lines, the database of fuzzy complexes, FuzDB, compiles experimentally observed fuzzy protein complexes, in which intrinsic disorder is maintained upon partner interaction, directly impacting biological function (10). An instructive example of this behavior is the extremely tight and functional interaction between the disordered histone H1 and its chaperone prothymosin-α, which retain their highly dynamic, fully disordered state in that complex (11). Structural interpretation of such a behavior may be assisted by data in the Protein Ensemble Database (PED) that encompasses experimentally determined structural ensembles of IDPs/IDRs (12).

There appears to be a consensus that these and other types of data and approaches will act in synergy to drive the field forward (13), toward reaching a better structural-functional understanding of the ‘disorderome’. Combined with data in the MobiDB database, which provides predictions and annotations for all IDPs/IDRs (14), this effort is also critical for integrating disorder-related information into other data resources, such as UniProtKB (15) or PDBe (16).

Not surprisingly, the field is also getting strong impetus from traditional structural biology: as missing regions from solved structures constitute a good proxy for structural disorder, improvements in structure determination techniques boost the identification of structural disorder. Cryo-electron microscopy has now advanced to a state that yields structures at atomic resolution (17). Similarly, the recent success of computational structure prediction by AlphaFold 2 (18) in overtaking the Critical Assessment of Structure Prediction competition, CASP14 (19), cannot be dismissed. AlphaFold's 2-based structure predictions of the entire human proteome (20) resulted in ‘only’ 58% of residues confidently covered with predicted structures, suggesting that structural disorder may be even more prevalent in proteomes than previously thought (21). Whereas it remains to be confirmed if certain AlphaFold 2 metrics can be harnessed for disorder prediction (20), it is without doubt that these achievements will help us navigate further through the ‘dark proteome’ (22).

Disordered proteins are often involved in disease, yet they represent a largely unexplored target for drug development (23,24). Recent successes in the field, i.e. targeting c-Myc (25), androgen receptor (26) or alpha-synuclein (27), encourage us to adhere to this ambition. The concept of liquid-liquid phase separation (LLPS) leading to the formation of membraneless organelles (MLOs) raises hope that IDP/IDR function and therapeutic targeting can now be approached from a novel angle (28,29).

To reflect the steady progress of the protein disorder field, it is important to update and upgrade DisProt as one of the primary resources of manually curated, experimentally confirmed protein disorder. The previous release of the database, DisProt 8 (30) contained about 1500 entries and 3500 disordered protein regions. In the current release, DisProt 9, not only did we increase these numbers, but also improved the reliability of entries by introducing a reviewing process. In addition, a great effort was allocated to training activities by providing DisProt biocurators with detailed curation guidelines and virtual training sessions and a published protocol describing how to explore manually curated annotations in DisProt (13), alongside the dissemination of new database content in a dedicated DisProt blog (https://disprot.org/blog/) and Twitter account (https://twitter.com/disprot_db). DisProt 9 presents a new graphical interface and updated features, such as the integration of two ontologies and the connection with APICURON, a database to credit and acknowledge the work of biocurators (31). With these improvements, DisProt continues to be a primary resource of protein disorder for the structural-molecular biology community.

## PROGRESS AND NEW FEATURES

### Database content

DisProt 9 includes 2038 protein entries and 4,477 pieces of evidence of state transitions, interactions and functions, featuring a 30% increase over the last release (DisProt 8), along with 2578 publications annotated, accounting for a 28% increase compared with DisProt 8. In addition, about 14% of the annotations, corresponding to 237 entries, have been reviewed and validated by an expert biocurator, e.g. the High mobility group protein HMG-I/HMG-Y (DP00040) has been thoroughly reannotated by revising spurious annotations and integrating new functions, such as *DNA bending* (GO:0008301, IDPO:00514), *DNA binding* (GO:0003677, IDPO:00065)*, RNA binding (*GO:0003723, IDPO:00066) and *protein binding* (GO:0005515, IDPO:00063).

In DisProt 9, annotations of amino acid repeats displaying the typical properties of IDPs, specifically poly-glutamate (polyE), poly-lysine (polyK) and poly-arginine (polyR) regions, have been added based on indirect evidence. These regions are always disordered but experimental evidence is provided for only a handful of cases or for engineered fragments. For example the polyE repeat in the PEVK region of human titin has a Stokes radius 2–4 times larger than expected based on its molecular mass, and it shows a minimum at 200 nm in its circular dichroism (CD) spectrum (32). In order to be included in DisProt a repeated region must be predicted by MobiDB-lite (33) (and be available in MobiDB) as a negative or positive polyelectrolyte subregion (14,32) and be at least 10 residues long. In DisProt 9, such cases are highlighted by a specific evidence code, *curator inference from database* (ECO:0007636).

**Figure 1.**
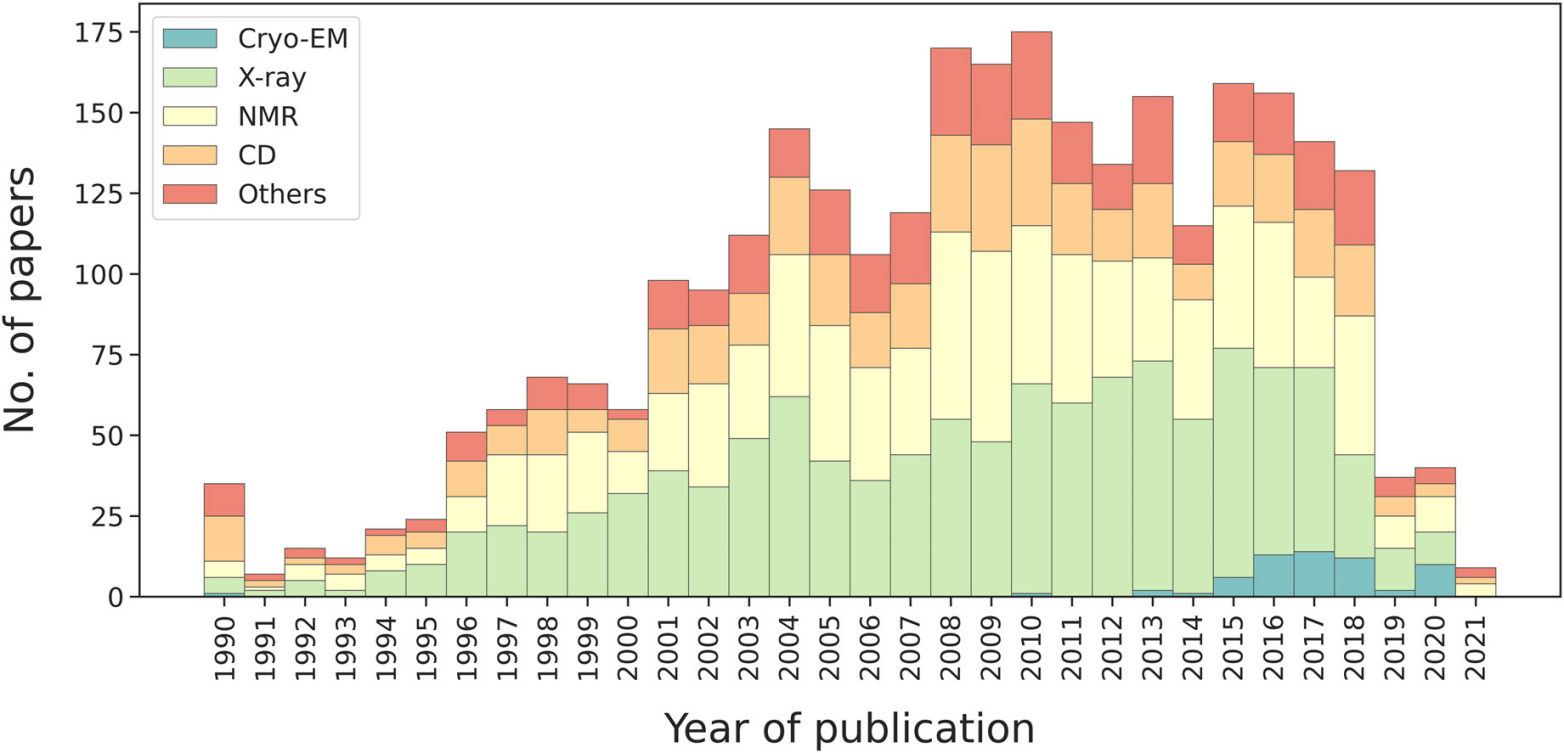
Number of experimental evidence of disorder used in DisProt by year of publication. Different colors correspond to different experimental techniques as reported in the corresponding publications. Publications older than 1990 are grouped in the first bar.

The distribution of regions based on the experimental detection method is shown in Figure 1. In accordance with the recent improvements that recognize cryo-EM as a well-established technique in structural biology (17), DisProt includes a total of 149 IDRs annotated by this method.

**Figure 2.**
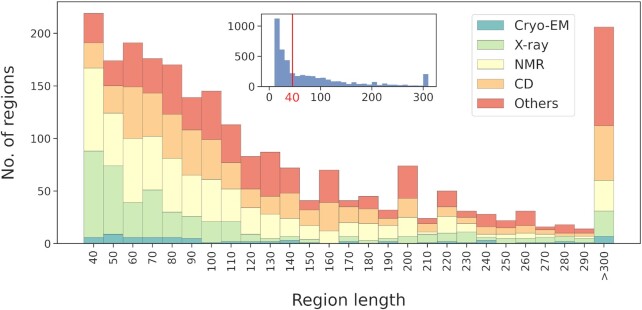
Region length distribution of pieces of evidence inferred using various experimental techniques in DisProt. Data in the main plot starts at length 40, the full distribution is shown in the inset plot.

Figure 2 shows the length distribution of regions annotated using a specific experimental technique. Atomic resolution techniques such as X-ray (ECO:0006187) and NMR (ECO:0006252) dominate the experiments used to characterize short (<100 residues) disordered regions, while other complementary methods and far-UV CD (ECO:0006179) are the techniques mostly used in detecting and characterizing longer IDRs.

**Figure 3.**
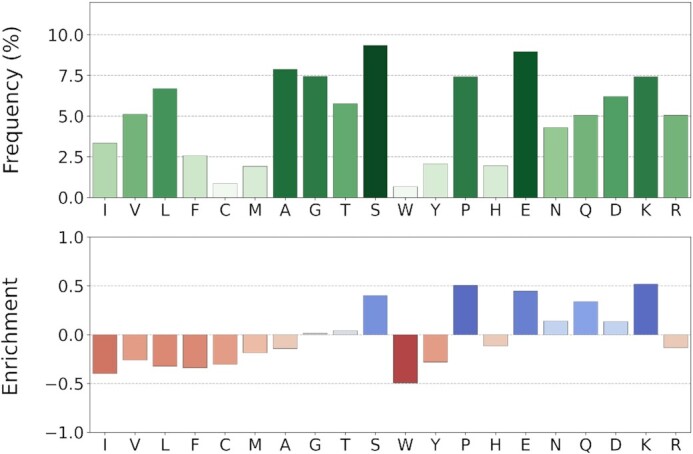
Amino acid composition of DisProt regions. Amino acids are sorted by the Kyte-Doolittle hydrophobicity scale. The amino acid frequency is calculated considering only disordered residues. The enrichment is calculated and normalized over the TrEMBL database frequencies (release 2021_03). Color intensity is proportional to bar height.

Figure 3 illustrates the amino acid composition and fold increase (enrichment) of DisProt proteins, as compared to TrEMBL distribution (release 2021_03). The fold increase, which is the difference between DisProt and TrEMBL frequencies normalized by TrEMBL, highlights amino acids which are over- and under-represented in DisProt IDRs. DisProt regions are enriched in disorder-promoting residues (Q, K, P, E, S), mostly charged and hydrophilic, while the hydrophobic, order-promoting residues (F, A, V, I, L), as well as arginine (R) are depleted and aromatic residues (W, Y, F) are strongly depleted. This amino acid distribution is in line with previously published results (34,35).

### Thematic datasets

Starting 2020, DisProt periodically releases ‘*thematic datasets*’ describing biological areas where IDPs play a crucial role. The first two thematic DisProt datasets illustrate the role of intrinsic disorder in *unicellular toxins and antitoxins* (December 2020) and *extracellular matrix proteins* (June 2021), by collecting carefully curated examples of IDPs and IDRs involved in the toxin-antitoxin system and in the extracellular matrix composition and function, respectively. DisProt thematic datasets are easily accessible from the DisProt home page under the ‘*Datasets*’ section. All the entries included in these datasets are tagged with the name of the *theme* and it is possible to download them as single files.

### Ontology

In DisProt 9, the previous Disordered Ontology (DO) has been renamed Intrinsically Disordered Protein Ontology (IDPO) and has been completely refactored. The ‘experimental method’ branch has been mapped one-to-one to a subset of the Evidence and Conclusion Ontology (ECO) (36), which was recently updated with new disorder-specific terms, e.g. IDPO:00125 maps to the corresponding *small-angle X-ray scattering* (ECO:0006182) ECO term. In addition, several new non-IDP-specific terms were introduced to cover a plethora of missing techniques, e.g. bait-prey protein pull-down evidence (ECO:0006249) is widely used to assess interactions and functions of IDPs and IDRs.

Similarly, all interaction terms and some function terms have been mapped to the Gene Ontology (GO) (37–39). Diverse modes of action of IDPs have been completely revised to provide the highest possible level of detail in the characterization of disorder functions. Despite GO being designed to annotate the whole protein, several of its terms are well suited to describe IDR behaviour, in particular those in the molecular function branch. IDPO terms were merged, mapped to existing GO terms or directly integrated into GO, thanks to an active collaboration between the DisProt and GO consortia.

All active functions, which exert their effect via molecular recognition on another interactor, are now annotated using GO terms, while self-regulatory functions (self-regulation or self-assembly via protein interactions in *cis)* and functions directly arising from the disordered state (*entropic chains*) are annotated by specific IDPO terms since corresponding terms are not yet available in GO. *Entropic bristle*, *entropic spring* and *entropic clock* terms have now been merged under the *entropic chain* term. The *Flexible N-terminal tail* and *flexible C-terminal tail* terms were introduced to highlight the presence of disorder in protein terminal regions.

The DisProt 9 website incorporates a new dedicated page for each IDPO term, e.g. https://disprot.org/idpo/IDPO:00501 for the *entropic chain* term. IDPO term pages include the identifier, name and definition of the term, its relationships with other terms and, when available, cross-references to external ontologies, e.g. Gene Ontology. Moreover, they list all entries annotated with that term.

The adoption of stable and well established ontologies, GO and ECO, plays a crucial role by allowing the curators to effortlessly expand the coverage of functions and experimental techniques available in DisProt. Since its partnership with the GO and ECO consortia, DisProt has become active in the definition of new terms that fulfill ontology-specific rules and constraints. Requests for new terms coming from the IDP community are welcome but must undergo GO and ECO approval. In the long run, all terms used by DisProt will be mapped to well established ontologies.

### Experimental ambiguity and MIADE

A number of DisProt annotations come from experiments performed under extreme conditions. These annotations, which were tagged as ambiguous in the previous DisProt version, are now described using the Minimum Information About Disorder Experiments (MIADE) guidelines (https://www.psidev.info/groups/intrinsically-disordered-proteins) (40). MIADE defines the minimal fundamental parameters that unambiguously characterize a disorder-related experiment. With MIADE, it is possible to evaluate and compare experimental evidence coming from other resources adopting the same standard objectively.

In DisProt 9, MIADE is implemented by including the following annotation fields: (i) *sequence construct* features, e.g. the exact sequence from the experiment along with its modifications, such as PTMs and mutations, (ii) the *experimental conditions*, i.e. pH, temperature, pressure and redox potential and (iii) the *experimental components*, e.g. small-molecules and membranes, along with values, deviation and additional variables. Further specifications are provided in the help page on the DisProt website.

### Biocuration and APICURON

DisProt annotations are provided by both professional and community biocurators. The whole community of biocurators is supported by a team of senior biocurators who check and validate annotations and deliver training material including face-to-face activities and a detailed curation manual. In order to properly attribute the curation effort, DisProt is now connected to APICURON (31). APICURON collects and tracks biocuration events from manually curated resources and implements gamification concepts, i.e. badges, medals and leaderboards, to promote biocurator engagement. The DisProt website integrates an APICURON widget (https://disprot.org/release-notes) which provides the ranking, number of activities and scores of the curators. In APICURON, all DisProt biocurators have a dedicated profile page that groups all the achieved badges and medals, along with their curation activity.

Additional annotations can be provided directly by the DisProt users through a ‘Contact us’ page. In DisProt 9, along with the ‘*Leave a comment*’ tab for feedback on the site experience, a new ‘*Submit a new annotation*’ section has been added.

### Implementation

DisProt 9 adopts the Document Versioning Pattern to precisely track all changes between different versions of the same record. This improves reproducibility, simplifies tracking of curation activities and allows the user to compare different versions of an annotated entry directly on the web site. Each protein has a *history* page and each IDR has an assigned version number. Whenever a curator modifies an existing region, the version counter increases by one.

DisProt exposes disordered region descriptions with Bioschemas metadata, including disordered region boundaries and type of disorder. The information content from the entry pages is aggregated together with complementary resources (MobiDB and PED) to the IDPcentral registry and knowledgebase (41).

The DisProt web application source code underwent a major upgrade, updating Angular version 12 and transitioning to Bootstrap version 5. The DisProt interface is now more intuitive, thanks to a minimalistic, consistent design between different components. The upgraded curation interface allows for more efficient work from biocurators, while raising the standards of curated data quality thanks to real-time syntax checks and cross-validation with various third-party data resources.

### Outreach activities

DisProt has an active social media presence on Twitter (https://twitter.com/disprot_db). This account promotes communication with users and experts in the field. It provides updated information about the latest release, webinars and training activities. Finally, a dedicated blog website (https://disprot.org/blog) contains descriptive extended posts about thematic datasets, general statistics and novel, interesting annotations. Training material for the users is provided via a published protocol describing how to explore manually curated annotations in DisProt (13). Training material for the curators is offered via recorded webinars and an updated user manual.

## CONCLUSIONS AND FUTURE WORK

DisProt is the gold standard for IDP/IDR annotations, and serves the community as a fundamental resource that drives biological hypotheses, experimental design and the training and benchmarking of disorder and function predictors. Compared to the previous version, it has improved data accessibility and quality, and significantly increased annotation volume. The content is updated frequently and is now more focused on function. A team of expert reviewers validate annotations provided by the community and continuously check the literature for novel experimental evidence.

DisProt is well connected to other databases and consortia, and is active in the development of new standards and ontologies. The *Intrinsically Disordered Proteins Ontology* (IDPO) has been refactored and systematically cross-referenced with *Gene Ontology* (GO) and *Evidence and Conclusion Ontology* (ECO). Experimental setup is now captured in an unambiguous and structured way by implementing the *Minimum Information About Disorder* (MIADE) standard.

The DisProt technological infrastructure has been renewed to improve reproducibility by implementing exhaustive versioning of all entries. The community of curators is engaged, structured, well trained and continuously updated. Finally, DisProt is now connected with APICURON to provide live tracking and proper attribution of the curation effort.

DisProt is committed to the reduction of false negative annotations, resolution of inconsistent annotations (e.g. between close homologs) and growth of functional descriptions. At the consortium level, DisProt is active on the definition of MIADE guidelines, creation of a standard controlled vocabulary to define conformational states (in collaboration with the structural biology community). From a technological point of view, DisProt is working to meet the format standards to export its annotations to core data resources such as GO, PDB, IntAct and UniProt. The long-term maintenance of DisProt is guaranteed by its central role within the European Union's Horizon 2020 IDPfun program and the ELIXIR IDP Community, the reference scientific communities involved in the study of intrinsically disordered proteins.

## DATA AVAILABILITY

The data that support the findings of this study are openly available in DisProt at https://disprot.org/.
